# Additive Biomanufacturing with Collagen Inks

**DOI:** 10.3390/bioengineering7030066

**Published:** 2020-07-01

**Authors:** Weng Wan Chan, David Chen Loong Yeo, Vernice Tan, Satnam Singh, Deepak Choudhury, May Win Naing

**Affiliations:** 1 Biomanufacturing Technology, Bioprocessing Technology Institute (BTI), Agency for Science, Technology and Research (A*STAR), Singapore City 138668, Singapore; chan_weng_wan@bti.a-star.edu.sg (W.W.C.); david_yeo@bti.a-star.edu.sg (D.C.L.Y.); vtan026@e.ntu.edu.sg (V.T.); Satnam_Singh@bti.a-star.edu.sg (S.S.); 2 Singapore Institute of Manufacturing Technology (SIMTech), Agency for Science, Technology and Research (A*STAR), 2 Fusionopolis Way, #08-04, Innovis, Singapore City 138634, Singapore

**Keywords:** collagen, ECM, extracellular matrix, bioinks, biomanufacturing

## Abstract

Collagen is a natural polymer found abundantly in the extracellular matrix (ECM). It is easily extracted from a variety of sources and exhibits excellent biological properties such as biocompatibility and weak antigenicity. Additionally, different processes allow control of physical and chemical properties such as mechanical stiffness, viscosity and biodegradability. Moreover, various additive biomanufacturing technology has enabled layer-by-layer construction of complex structures to support biological function. Additive biomanufacturing has expanded the use of collagen biomaterial in various regenerative medicine and disease modelling application (e.g., skin, bone and cornea). Currently, regulatory hurdles in translating collagen biomaterials still remain. Additive biomanufacturing may help to overcome such hurdles commercializing collagen biomaterials and fulfill its potential for biomedicine.

## 1. Introduction

Collagen is by far the most prevalent extracellular matrix (ECM) molecule found in adult mammals with an estimated 30% of protein mass of multicellular organisms [1]. Although the collagen molecule has 29 subtypes (variants) [2,3], approximately 90% of collagen consists of variants types I, II, III [4]. Collagen extracellular matrix can be found throughout the body in both soft and hard connective tissues including bones, skin, tendon, cartilage, cornea, lung, liver etc. [5].

**Figure 1:**
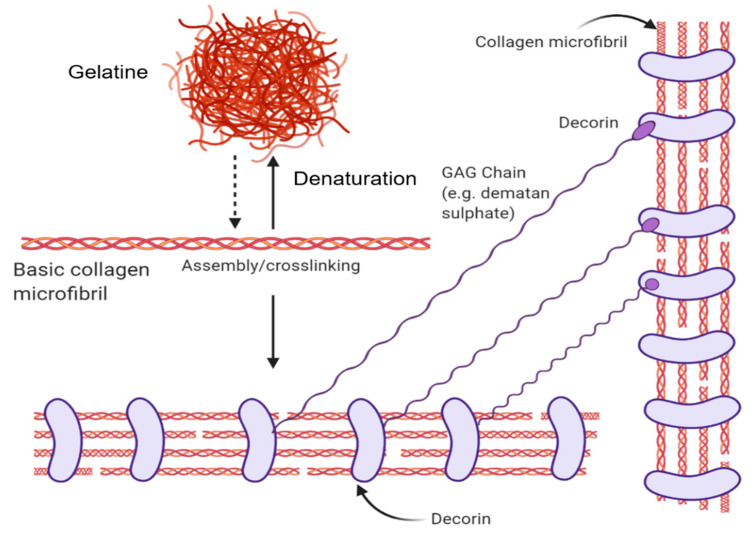
The structural forms of collagen and their native interactions. The basic collagen unit is a triple-helix microfiber that denatures into gelatine or can be assembled into collagen fibrils. Decorin proteins wrap around collagen fibrils in their native context and bind with glycosaminoglycan chains such as dermatan sulphate. Created with BioRender.com.

Its fundamental structural unit is a 300 nm protein consisting of 3 braided α-subunits of 1050 amino acids in length. Each strand comprises the repeating amino acid motif: Gly-Pro-X (X is any amino acid). These strands form hydrogen bonds between the NH bond of a glycine and a carbonyl (C=O) group from an adjacent strand that holds the structure together and form their characteristic triple helix structure [4,6]. Collagen is a hierarchical biomaterial that is self-assembled into fibrils (containing numerous structural units) of ~1 cm length and ~500 nm in diameter (using type 1 Collagen as the archetype). Fascinatingly, the individual tropocollagen monomers are unstable at body temperature and favour random coil conformations. However, collagen fibrillogenesis gives rise to triple helix macromolecular structures with favourable mechanical strength in 3-dimensions, with resistance to enzymatic degradation [6]. Through the introduction of energy (e.g., heat energy from the surroundings), the H-bonds maintaining the orderly collagen structure are separated, causing the individual strands of the triple-helix to separate, resulting in a disorganized, denatured state known as gelatin (please see Figure 1 for more information).

The Gly-Pro-X amino acid arrangement is critical to the collagen molecule as seen from disease-causing mutations that lead to osteogenesis imperfecta or “brittle bone” disease. A single misplacement of glycine due to the mutation results in unstable helices [4]. In their native microenvironment, collagen molecules interact with other biological molecules. Negatively-charged Glycosaminoglycans (linear polysaccharides) sequester growth factors within the ECM [7]. These have been used to generate bio-active collagen scaffolds for cell growth [8]. Furthermore, Collagen interacts with Elastin fibers to provide recoil to the ECM, as well as fibronectin to mediate cell attachment and function [1]. Collagen molecules can also interact with reducing sugars in the body which result in its glycation. Glycation molecules result in the formation of advanced glycation end products (AGEs) which gives rise to the loss of soft tissue biomechanical properties and is associated with various diseases such as atherosclerosis, osteoporosis, diabetes and renal failure [9].

Collagen biomaterials have been utilised for decades to enhance cell culture/function [10]. A number of collagen or collagen-derivative based protocols and commercial culture products have been used extensively ranging from cell culture surfaces to hydrogels [10]. These include culture well inserts [11,12] (MilliCell^®^, Transwell^®^), sponge/gels (Matrigel™, Extracel™) and microcarriers (GEM™). While matrigel is derived from Engelbreth–Holm–Swarm (EHS) tumor and found to contain collagen IV, laminin and heparin sulfate, GEM ™ microcarriers coat an alginate core with gelatin to aid cell attachment.

Beyond cell culture reagents, collagen biomaterials have been used for tissue engineering applications including: bone, tendon, cardiovascular therapies and disease models [13], cornea [5], skin, skeletal muscle, artery [14] etc. One usage with great popularity is using collagen scaffolds as dermal regeneration templates for severe wounds and other trauma such as burns. To date, a number of scaffolds/templates containing collagen ingredients are commercially available including: Helistat (Integra ^®^), Instat (Johnson & Johnson), SkinTemp (BioCor), Helitene (Integra ^®^), Fibracol (J&J), Biobrane (UDL Laboratories), and Chronicure (Derma Sciences)–not an exhaustive list, which is currently presented in fibre, powder, composite forms etc. [15]. Collagen biomaterials as dermal templates have seen the greatest number of commercial translations to date. Recently, novel applications in sustainable cellular agriculture using collagen biomaterials include making artificial leather and bio-artificial muscle [16].

Despite plentiful collagen biomaterial applications developed, collagen has several limitations that curtail its widespread usage: generally poor mechanical properties (vascular tissue engineering applications), thrombogenicity, contamination, source and batch variability [13]. These limitations leave many collagen biomaterial applications in the earlier technology development stages, hindering technology translation. 

The emerging field of biomaterials printing - bioprinting, provides the means to create structures from collagen biomaterials, additives and cells in a reproducible and scalable way [17,18]. Adapted from methods first used to manufacture inorganic materials [19], bioprinting is an additive manufacturing approach to produce living tissue and organ analogs for regenerative medicine, tissue engineering, pharmacokinetic and disease/developmental modelling [20]. By patterning various combinations of biomaterials and cells, a goal is to reproduce complex biological architecture to recreate the anatomy in reproducible ways [21,22]. Thus, bioprinting potentially mitigates concerns of product variability by increasing process reproducibility. Moreover, increasing production throughput with bioprinting circumvents bottlenecks in production capacity, making collagen biomaterial products more cost-effective. 

This article focuses on the bioprinting of collagen biomaterials/bioinks for (mostly) therapeutic purposes. Bioinks differ from biomaterials in that cells are introduced with the materials and printed, even in situ [23]. On the other hand, biomaterial scaffolds are printed alone before cellular components are added. We discuss how collagen biomaterials are isolated from different sources, processed and analysed post-processing. Thereafter, we discuss various printing methods for collagen biomaterials ranging from manually-casted production (the simplest and lowest throughput) to stereo-/digital light printing (additive manufacturing suited for producing complex shapes). The article concludes with a discussion about translational regulatory, cost and strategy issues using bioprinted collagen biomaterials/bioinks for regeneration and therapy applications. 

## 2. Processing Parameters

Each step in the processing of collagen for additive manufacturing alters the properties and structure of collagen. Depending on the sources of collagen, extraction steps and crosslinking methods (chemical, physical), the resultant properties will differ. The effects of these processes as well as methods for analyzing collagen biomaterials will be discussed.

### 2.1. Sources of Collagen

For additive biomanufacturing, fibril-forming sub-types of collagen (type I, type II, type III, type V, type XI, type XXIV and type XXVII) are preferred because they contribute to the mechanical integrity of the ECM [15,24]. Fibrillar collagen is formed from the assembly of collagen molecules because of the intermolecular bonds between the individual strands to create the signature triple-helix collagen molecule (see the introduction section). These fibrils further assemble into fibre-bundles with tensile strength in tendons and skin [3] or into orthogonal transparent layers (e.g., cornea) [25]. 

Fibrillar collagen can be extracted from various sources. As animal skin/tendons and cartilaginous tissues are abundant in type I and type II collagen respectively, these tissues are sources of fibrillar collagen extraction [26]. Cells cultured in vitro are used to synthesize collagen as well [27,28]. Cells such as fibroblast and chondrocytes which specialize in type I and type II collagen production respectively can be cultured and the synthesised collagen harvested from media or cell layers. Recombinant collagen production is using genetically engineered microorganisms, plants or animals such as bacteria, yeast, transgenic corn and silkworms [29,30]. Synthetic peptides mimicking collagen trimeric structure have also been investigated to produce collagen-like peptides [31,32]. Collagen from cells grown in vitro, recombinant protein production as well as peptide synthesis have very low yield and are not as cost-effective as collagen extraction from animal tissues. Hence, most commercial collagen extraction relies on animal sources. While there are variations in collagen between different animal species and tissue sources, variation of collagen exists as well, within the same species due to the nature of collagen. As the collagen molecules in animals form mature crosslinks over time, the age, gender, activity and physical state of the animals play a significant role in forming these crosslinks [2]. The variability of collagen between batches of extraction affects fibrillation and self-assembly properties, and in turn the final collagen biomaterial product.

### 2.2. Collagen Extraction

Collagen extraction depends on its solubility in the chosen solvent and composition of collagen types in the tissue sources [26]. Collagen extraction can be broken down into 3 stages: Pre-treatment, extraction and purification. During the pre-treatment step, non-collagen proteins are removed to increase the yield of the collagen extraction process. Depending on the tissue source, removal of the non-collagen proteins (lipids, calcium, etc.) is achieved using alkali solutions, neutral saline solutions, alcohol solutions or a combination of solution [33]. Following pre-treatment of tissues, collagen is then extracted via acid-solubilisation or enzymatic-digestion.

In the extraction of collagen by acid-solubilisation, the pre-treated tissue is added into a dilute acidic solution, typically acetic acid, to disrupt weaker hydrogen bonds between collagen molecules [26]. This allows tissue swelling and acid-soluble collagen (ASC) from the loosened structure to dissolve in dilute acid [34]. However, dilute acid does not disrupt the triple helix structure of collagen due to the strong intermolecular forces between the polypeptide strands [35]. The extracted collagen still retains its telopeptide region and is known as telocollagen.

In the extraction of collagen by enzymatic-digestion, pre-treated tissue is added into a proteolytic enzyme solution, typically pepsin which cleaves non-helical telopeptide at the ends of the collagen microfibrils. Selective cleaving of the telopeptide region results in the destabilisation of the fibril structure and increases collagen dissolution [34]. The triple helix structure of collagen is unaffected due to the selective pepsin enzyme digestion. The extracted collagen molecule does not retain its telopeptide regions and is known as atelocollagen.

While clinical use of collagen use both telocollagen as well as atelocollagen in dermal substitute product showed no collagen induced adverse immunogenic response, the removal the telopeptide regions is suspected to play a role in the immunogenicity and antigenicity of collagen [36]. This is because the immune response in the body targets the antigenic determinant are found in mostly the telopeptides of collagen [37]. However, the antigenic determinants which arise from the helical structure and the amino acid sequence of the collagen also contribute to the immunogenicity and antigenicity of collagen [37]. Additionally, antigenic determinates for immune responses in the body depends on the species as well [36].

These extraction methods are not exclusive and can be performed together. Enzymatic-digestion can be done on acid insoluble collagen to obtain higher yields [26]. The extracted collagen is then filtered to remove impurities and purified through repeated salt precipitation, centrifugation and dissolution in acetic acid. Alternatively, the filtered extract undergoes dialysis for purification before freezing and freeze-drying.

#### 2.2.1. Various Forms–Native, Gelatin (Disordered), Collagen Peptides

Depending on extraction methods used, the molecular weight, α-chain composition, and molecular structure are affected, in turn resulting in a change to the properties of the collagen (e.g., solubility, viscosity, etc.) From the extraction process, collagen can further be processed into denatured forms. Using thermal energy, acids, enzymes or a combination of methods, the intramolecular bonds between the α-chains are broken. As a result, the native helix structure transforms into a random coiled structure known as gelatin. Gelatin is formed as a result of the hydrolytic cleavage of collagen into individual protein strands [34]. Further processing of gelatin into smaller peptide chains is achieved through proteolytic enzymes resulting in hydrolysed collagen. Hydrolysed collagens molecular weight is significantly smaller (3–6 kDA) compared to their native structure (~300 kDa) [38]. As a result, hydrolysed collagen is much less viscous and more soluble than its native counterpart. For this review, we will only limit our discussion to collagen-based inks for bio-additive manufacturing. While collagen is favoured for its excellent biocompatibility, it exhibits poor mechanical properties [34]. This limitation can be overcome by crosslinking collagen molecules which will be discussed later (Section 2.3 Methods of Collagen Crosslinking). 

#### 2.2.2. Collagen Biocomposites

To enhance/modify the biological and mechanical properties of collagen, a mixture of synthetic or natural polymers are used. Blending of collagen together with synthetic polymers gives the final product enhanced mechanical and biological properties. The use of biocompatible synthetic polymers such as poly(lactic acid) (PLA) and polycaprolactone (PCL) allow products with excellent mechanical properties [39,40]. These composites have both the beneficial biological properties of collagen and the mechanical stiffness of synthetic polymers. Blending collagen with natural polymers (such as hyaluronic acid [41], alginate [42], glycosaminoglycans (GAGs) [43], growth factors [44], etc. [15]) for biomaterials that better mimic the native ECM environment or elicit a desired cellular response. Other than natural and synthetic polymers, inorganic compounds such as hydroxyapatite [45] or Tricalcium Phosphate (TCP) [46] can be incorporated to elicit desired cellular responses as well. 

### 2.3. Methods of Collagen Crosslinking

Additional crosslinking of collagen molecules can be used to enhance the mechanical properties of collagen to provide structural integrity for additive bio-manufacturing such as for muscle tissue (8–20 kPa), cartilage tissue (20–30 kPa) and bone tissue (2–30 GPa) [45,47]. These “artificial” crosslinking bonds can be generated using chemical agents or physical treatment. Increasing concentration of chemical and treatment times generally increase collagen crosslinking. However, when using chemical agents for crosslinker, residual unreacted chemicals and/or chemical byproducts are often left behind [15]. This needs to be managed by washing to minimize cytotoxicity.

#### 2.3.1. Chemical Crosslinking

A commonly used aldehyde for collagen crosslinking is glutaraldehyde (GA). As a dialdehyde, the crosslinker reacts with available amide groups on the collagen chains via Schiff base reactions resulting in covalent imide linkages [48]. These covalent linkages stabilise the intramolecular and intermolecular collagen structure. However, unreacted GA is cytotoxic as it crosslinks cellular proteins which disrupt cellular functions, causing cytotoxicity. GA is used in varying concentrations (0.0025–2.5% wt/v) and treatment times (20 min to 24 h) [49,50,51,52,53,54,55]. Increasing concentration and treatment times lead to increased collagen crosslinking.

Carbodiimides can also be used for collagen crosslinking such as 1-ethyl-3-(3-dimethylaminopropyl) carbodiimide (EDC). EDC crosslinks the amino and carboxyl groups collagen in a 2-step process: EDC first activates the carboxyl groups of collagen, the activated group then forms an amide linkage with primary amines in collagen [56]. This results in zero-length crosslinking where covalent bond formed is directly between the amino and carboxyl groups without addition of EDC. Crosslinking stabilises the intramolecular and intermolecular collage structure, improving overall mechanical stiffness of collagen as well as the bending stiffness of collagen fibrils. Typically, the use of EDC is accompanied with N-hydroxysuccinimide (NHS), which allows a higher conversion of crosslinks due to amine-reactive intermediates stabilizing [57]. EDC or EDC together with NHS are used in varying concentrations (0.01–2.5% wt/v) and treatment times (2 h to 48 h) [46,51,54,55,56,58,59,60,61,62]. Increasing concentration and treatment times lead to increased crosslinking of collagen.

Hexamethylene di-isocyanate (HDI), an isocyanate is also used for crosslinking as HDI reacts with available amide groups on the collagen in a nucleophilic addition reaction [63]. The resultant reaction forms a urea linkage to stabilize the intramolecular and intermolecular collage structure [64]. HDI was used in varying concentrations (1.5–5%) and treatment times (5 h–overnight) [55,63,65]. Increasing concentration and treatment times lead to increased crosslinking of collagen.

Plant extracts such as tannic acid and genipin have been explored as sustainable crosslinking agents as well. Tannic acid (TA) is a polyphenol extracted from plants which stabilises the intermolecular bonds of collagen via hydrogen bonds and hydrophobic interactions between TA and collagen molecules [66]. Tannic acids of varying concentrations (0.1% to 6% wt/v) and treatment times(10 min to 120 h) [66,67,68]. Increasing concentration and treatment times lead to increased collagen crosslinking. Genipin is an Iridoid glycoside compound extracted from plants able to crosslink the free primary amines in protein [69]. This allows genipin crosslink primary amides in collagen, stabilizing the intramolecular and intermolecular collagen structure. Genipin is used in varying concentrations (0.00025% to 0.6%) and treatment times (1 h to 48 h) [69,70,71,72].

#### 2.3.2. Physical Crosslinking

The use of chemical crosslinkers inevitably faces issues with cytotoxicity. Physical methods such as dehydrothermal (DHT) treatment and ultraviolet (UV) irradiation are used to create covalent bonds between intermolecular collagen structures. 

Dehydrothermal treatment is a thermal treatment process that subjects collagen to high temperatures (>90 °C) for several hours or days (12 h to 5 days) under vacuum [40,51,54,73,74]. As a result, condensation reactions occur: between the free amino and hydroxyl groups of collagen (esterification); or between the carboxyl and free amino groups (amide linkage formation) [73]. These ester and amide bonds stabilise intramolecular and intermolecular collagen bonds. Despite the low water content of the collagen in vacuum, due to high temperatures, hydrolysis of the peptide bonds occurs resulting in the collagen triple-helix structure denaturing [73]. Though the mechanical properties of collagen improve with longer treatment times and higher temperature, collagen denaturing increases as well. 

UV crosslinking involves irradiating collagen (15 min to 240 min) [74]. The mechanism of crosslinking is a result of free radical formation from peptide bond scissions. UV irradiation forms aromatic radicals which in turn attack the peptide bonds in collagen. These radicals then interact and crosslink, which stabilises intramolecular and intermolecular collagen structure. The effectiveness of UV irradiation depends on the sample preparation, irradiation dose and time of exposure [75]. While UV irradiation improves mechanical properties, it also denatures collagen triple-helix structures [75].

Gamma irradiation crosslinking is similar to UV crosslinking where the collagen structure is irradiated for a period of time (250 min to 1250 min) depending on the desired irradiation dosage. Gamma irradiation “radio-lyzes” water, creating radicals. The effectiveness of crosslinking depends on irradiation dose and exposure time. Compared to UV irradiation, the higher energy of gamma irradiation is able to deeper penetrate thicker collagen structures. However, its downside is denaturing collagen’s triple-helix structure. Furthermore, gamma irradiation is often used for sterilization, making it unsuitable to crosslink cell-laden bioinks [76].

While cytotoxic compounds are not formed using physical crosslinking methods, they generally lead to collagen denaturation. Furthermore, physical crosslinking methods are less effective in improving mechanical properties of collagen compared to chemical methods [70].

### 2.4. Collagen Analytical Methods

Understanding the structural, morphological, and chemical composition of collagen is critical since additive bio-manufacturing processes may give rise to significant changes. Understanding the structural, morphological and chemical composition allows better design and processing of the collagen raw material to meet the needs of the final product [34,77].

#### 2.4.1. Structural Analysis

Differential Scanning Calorimetry (DSC) can determine collagen thermal stability. DSC compares and measures heat flow differences between a specimen and control when heat is supplied. Using this information, the denaturation temperature of collagen can be determined due to endothermic processes observed during collagen denaturation [78]. Using DSC, the denaturation temperature of soluble fish collagen was determined to be 10 °C lower than soluble porcine collagen [79].

Sodium Dodecyl Sulfate Polyacrylamide Gel Electrophoresis (SDS-Page) is used to visualise molecular size distribution of collagen protein fragments. SDS-Page uses an electric field to drive charged proteins through gel. Larger fragments move slower, while smaller fragments move quicker through the gel. Following separation by size, the fragments are stained with Coomassie blue or silver to obtain protein bands. Comparing these with controls of known molecular weight, can determine protein fragment weight. By comparing the banding pattern of known type I collagen chains (α1(Ⅰ): 97 kDa; α2(Ⅰ): 95 kDa), SDS-PAGE was used to determine the molecular weight of type V collagen chains through the relationship between relative molecular weight and migration rate [80].

Circular Dichroism (CD) is an absorption spectroscopy method to determine the presence of secondary and tertiary collagen structures. CD measures the differences in absorption of left circularly polarised light and right circularly polarised light. Due to the nature of the peptide bonds and structures, it results in characteristic absorption spectrums. From this information, the secondary and tertiary protein structures such as α -helices (negative bands at 222 nm and 208 nm; positive band at 193 nm), β-pleated sheets (negative band at 218 nm; positive band at 195 nm), triple helical conformation (negative band at 195 nm; positive band at 220 nm) can be determined respectively [81,82].

Raman spectroscopy is a label-free and non-destructive method used to determine the bonds and protein structures present in collagen. Raman spectroscopy measures inelastic light scattering of a sample from incident light generated by a laser source. The bonds and protein structures result in distinct shifts in wavelength of scattered light and hence distinct spectrum peaks such as Amide I band (1655 cm^−1^), Amide III band (1268 cm^−1^), α-helix shoulder (1630 cm^−1^) and β-pleated sheet peak (1675 cm^−1^). From this information, the relative quantities of bonds and protein structures can be determined for collagen [83].

FTIR is a spectroscopy method to determine the bonds and protein structures present in collagen. FTIR measures absorbance or emission of infrared radiation from a sample after irradiation from an infrared source. The bonds and protein structures result in distinct infrared spectrum peaks. Typical peaks of type Collagen are: Amide A (3299 cm^−1^), (N–H) stretching; Amide B (2919 cm^−1^), (CH_3_) asymmetric stretching; amide I (1628 cm^−1^), (C=O) stretching; amide II (1540 cm^−1^), (N–H) bending & (C–N) stretching; amide III (1234 cm^−1^), (–CONH_2_) stretching. From this information we can determine the presence of bonds and protein structures and their relative quantities in collagen [84]. Additionally, the ratio peak intensity of 1 between the amide III peak and 1450 cm^−1^ is indicative of the helix structure of collagen [84]. 

#### 2.4.2. Morphological Analysis

Scanning Electron Microscopy (SEM) uses focused beams of electrons to image surface topography of collagen samples. SEM measures the energies of elastic and inelastic-scattered electrons incident upon the sample to recreate surface topography. Typically, SEM can examine porosity of collagen sponges as well as assembled collagen fibre structures. SEM was used to study pore morphology of collagen sponge, collagen-I fibrin gel, collagen 2D nanofibers (oriented and random) [85].

Confocal microscopy can be used for structural visualization too. Confocal microscopy sections images for each focal plane using a laser source before compilation into a 3D image volume of high resolution. There are two modes of image acquisition: fluorescence [86] and reflectance [87]. Fluorescence image acquisition uses fluorescent dyes or autofluorescent properties of collagen to generate image contrast, while reflectance image acquisition relies on differences in refractive indexes. Collagen fibril diameters and pore sizes have been studied using both modes of acquisition [86,87].

Transmission electron microscopy (TEM) is a microscope used to visualise banded collagen fibril structures. TEM generates an image by transmitting an electron beam through a thin specimen on a copper grid, the image is then magnified and projected onto a stage. The regular array of gaps and overlaps in collagen microfibrils result in differences in packing density along the assembled collagen fibre. This leads to the banded structure of the collagen fibrils (64–67 nm). Cryo-TEM was used to analyse fibrillar collagen from mineralized and non-mineralized tissue [88].

Atomic force microscopy (AFM) also visualises banded collagen fibril structures. AFM generates an image by measuring deflection of a cantilever probe across collagen fibres. This information is then rendered into a topographic images. AFM is able to detect differences in packing densities that arise from the array of gaps and overlaps in collagen microfibres [89].

#### 2.4.3. Chemical Assays

Hydroxyproline is a colorimetric assay for quantifying hydroxyproline in collagen. Due to the hydroxyproline amino acid composition being approximately constant across the different types of collagen 11.3% (type I) and 15% (type III), it can indicate the amount of collagen within a sample [90].

Sircol assay is a colormetic assay to quantify collagen, binding to the [Gly-X-Y]_n_ helical structure in collagen. Collagen content can be obtained by comparing it to standard curves for calibration [91].

2,4,6-Trinitrobenzne sulfronic acid (TNBS) assay is a colorimetric assay used to quantify free primary amines found in collagen. The amount of free primary amino groups can be obtained by comparing it to known quantities. The amount of TNBS can determine the degree of collagen methacrylation [92].

Ninhydrin assay is a colorimetric assay to quantify free primary amino groups. The dye binds to primary amines found in collagen. It was used to determine the change of free amino groups on collagen nanofibers following pre-treatment of L-lysine [93].

Western blot is a method used to identify the type of collagen following SDS-page analysis. Using monoclonal antibodies specific to the collagen types and visualisation through immunofluorescent staining, the type of collagen can be identified. Western blot was used to confirm Collagen VI chains from cell extracts and culture media [94].

Mass spectroscopy identifies proteins from gaseous ions generated from the protein fragments. These are sorted using an electric field according to mass-to-charge ratio. The relative quantities of ions are recorded. By comparing profiles of protein fragments with a database, the proteins can be identified. Mass spectroscopy was able to identify crosslinked pyridinoline and deoxypyridinoline amino acid in hydrolysed collagen [95].

## 3. Collagen-Based Ink Printing Applications

The application of collagen-based ink in both non-additive and additive manufacturing requires understanding of collagen processing, as well as the various printing methods. In this section, the principles behind the printing methods and their applications are examined.

### 3.1. Non-Additive Manufacturing

Non-additive manufacturing methods, casting and electrospinning of collagen-based inks and their applications are discussed. Casting involves pouring a liquid material into a mold of desired shape before solidifying and removal. Typically for collagen-based biomaterials, highly porous 3D structures (sponges) are obtained via the freeze-drying process while thin-films are obtained via air drying [34]. Freeze drying is a complex process where ice crystals in the frozen mold are removed by sublimation under vacuum. Pore size and direction of the sponge can be controlled during freeze-drying [96,97].

Collagen sponges are used extensively in wound healing and tissue engineering as scaffolds for bone [98], skin and soft tissues [99]. The porous nature of collagen sponges allow cell migration as well as nutrient diffusion into the scaffold while providing a substrate for growth. The collagen sponge can be loaded with drugs, growth factors and bio-additives to enhance scaffold bioactivity [50,60,98,99,100]. Collagen-glycosaminoglycan scaffolds have been successfully used to regenerate skin from full thickness burns [50]. Additionally, by varying the glycosaminoglycan concentration and pore size, peripheral nerve tissue was successfully regenerated too [101]. Loading TGF-β1 into a collagen sponge allowed controlled release of growth factors, enhancing bone regeneration of a rabbit skull defect [44]. 

When collagen is laid out to dry, a thin-film of collagen is obtained via evaporation. As water and solvents evaporate, fibres and molecules are brought closer together due to surface tension of the solvent giving rise to a thin-film layer upon drying [34]. Thin collagen films are typically used in cornea treatment owing to their optically transparent nature and biological properties [61]. However, collagen films are not limited to ocular tissue engineering, micropatterns can also be designed onto the film as part of the casting process to influence osteoblast cell orientation [54]. By stacking the collagen film layer by layer, the resulting biomatrix encouraged neo-tissue formation in a hernia repair model [71]. The films can also be wrapped into tubes for nerve grafting applications [49]. While functioning as a barrier membrane, collagen films can also be loaded with drugs, growth factors and bio-additives to enhance bioactivity. Additionally, collagen film degeneration and its mechanical properties can be controlled by varying crosslinking to control the release of its contents via degradation [102,103]. Collagen films are also suitable as edible food packaging [104].

#### Electrospinning

Electrospinning consists of loading a desired biomaterial and a volatile solvent into a syringe. By applying a voltage to the needle tip, an electric field forms between the needle tip and the collector. Once, the electrostatic forces of repulsion are greater than the surface tension of the extruded liquid, a taylor cone is formed and the charged liquid is ejected onto the collector. The volatile solvent evaporates, resulting in fine nano/microscale fibres. These fibres are then deposited onto the metallic collector. By varying the extrusion rate, voltage of charged material, needle gauge and distance between the needle and collector the fibre diameters can be controlled [105]. 

Processing materials via electrospinning is appealing due to the ability to produce fibre meshes with diameters similar to the native fibrillar network present in the extracellular matrix (20 nm to 40 µm) [106]. Electrospinning can be performed using pure collagen or synthetic polymer additives such as PLLA or PCL to increase mechanical stiffness. Various electrospinning set-up can be used to produce different scaffolds for a variety of applications. A co-electrospinning system containing 2 mixtures of collagen and synthetic polymers was used to produce a scaffold with different regions to mimic muscle-tendon junction properties [107]. Using multi-layered electrospinning, an arterial structure was fabricated using a PCL, elastin and collagen layer was able to achieve significant improvement in mechanical properties and designed to mimic native arterial tissue [108]. A combination of electrospinning and electrospraying technology was used to produce 3D constructs which improved cell infiltration and controlled release of bio-additives [109].

However, the solvents used in electrospinning can significantly denature collagen. Typical fluoroalcohols used in electrospinning such as 1,1,1,3,3,3-Hexafluoro-2-propanol (HFP) cause a loss of collagen’s triple helical structure [106]. Fortunately, solvents have been designed to minimize collagen denaturation when electrospun using “less harsh” solvents such as acetic acid/DMSO and PBS/ethanol [110].

### 3.2. Additive Biomanufacturing

In this section, four additive bio-manufacturing technologies will be discussed: extrusion bioprinting, inkjet bioprinting, laser-assisted bioprinting and stereolithographic/digital light processing bioprinting. The main advantage of additive bio-manufacturing is to produce complex shapes with internal structures at high resolution and accuracy without molds or shaping tools required by non-additive methods. Moreover, additive bio-manufacturing is amenable to printing with cell-laden inks (bio-inks) [24].

While all additive biomanufacturing processes create structures via layer-by-layer deposition of biomaterials, not all collagen-based inks can be printed using the following methods. As such, flexible printing method such as extrusion printing have a larger number of applications and variation of printing formulations, whereas more restrictive printing methods such as inkjet, laser-assisted, and stereolithography printing have fewer applications.

#### 3.2.1. Extrusion

In extrusion bioprinting, biomaterial inks are loaded into a syringe and printed as filaments onto a stage via a mechanical or pneumatic dispensing system. Precise deposition of material is controlled by a dispensing stage along the x, y, and z axis. This method of bioprinting accommodates a large range of ink viscosities (30–60 × 10^7^ mPa∙s) [18]. Through multiple print heads, multiple materials and formulations can be printed together. However, this printing method is limited by the print resolution (100 µm), which is determined by the nozzle diameter [111]. Furthermore, printed cells experience high shear stresses when extruded under high pressure and small nozzles, resulting in lower cell viability.

Due to the nature of extrusion bioprinting, the viscosity of the collagen-based ink plays an important role in the printing process. The tendency of collagen to self-assemble into fibrillar structures at neutral pH when incubated at 37 °C allows collagen to form stable structures after printing [2]. Pure collagen was formulated to be self-supporting by either increasing the concentration or neutralising pH prior to extrusion. Following the extrusion process, scaffolds self-assembled in a neutral buffer to support self-assembly. This process produced tissue spheroid scaffolds as well as printing cell-laden inks into pre-set extrusion designs [47,112].

Combining collagen with other polymers, it is possible to design self-supporting structures by incorporating polymers rather than solely relying on pure collagen. An example was the use of cell-laden collagen/gelatin/alginate ink, by taking advantage of a two-step process involving thermal crosslinking with gelatin at low temperatures followed by crosslinking alginate in calcium solution [113]. The construct was printed at low temperature for gelatin to thermally crosslink and support the structure. Thereafter, it was immersed in calcium solution for ionic crosslinking of alginate to fix its shape. Gelatin and alginate was removed via diffusion and sodium citrate respectively, leaving behind a cell-laden collagen structure. A similar approach was used in cell-laden collagen/alginate ink where coaxial extrusion of collagen-alginate inks with calcium solution allowed the printed ink to be self-supporting [114]. In another, Pluronic F-127/Collagen ink was used to modify the gelation of the printed collagen ink, allowing it to be self-supported and be removed via diffusion in media [115]. A process unique to extrusion bioprinting known as freeform reversible embedding of suspended hydrogels (FRESH), non-self-supporting collagen ink formulations can print complex collagen scaffolds which are then self-assembled and collected from the hydrogel suspension [116].

**Figure 2:**
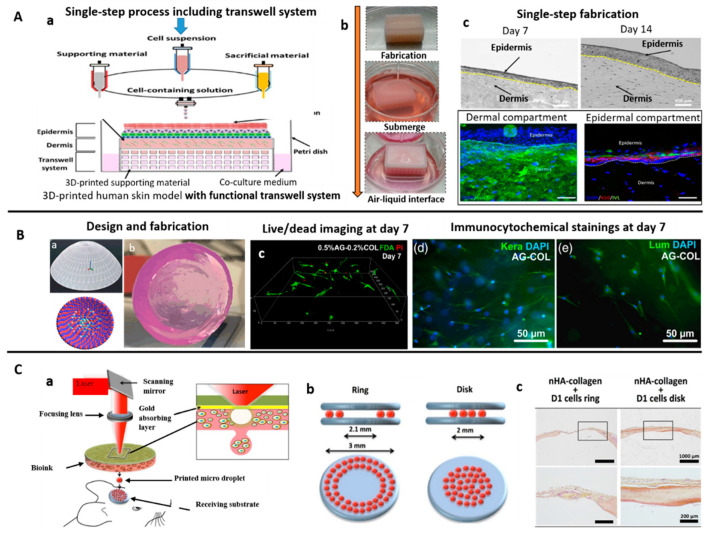
Bioprinting of collagen-based inks for tissue engineering. (**A**) (a,b) Hybrid system (extrusion-based and inkjet-based dispensing modules) used for bioprinting of collagen bioink for developing human skin models, (c) bioprinted model showed good structural features and respective dermis (Col) and epidermis (K10) biomarkers [118]; (**B**) (a,b) Drop-on-demand (DoD) bioprinting was used for bioprinting collagen bioink to develop functional biomimetic 3D corneal model, (c) 3D view of human CSK 7 days after bioprinting stained with live/dead staining, most of cells found viable, (d) Smooth muscle actin immunocytochemical stainings of CSK-loaded agarose-collagen blends 7 days after bioprinting, observed positive keratocan (Kera) and lumican (Lum) expression [120]; (**C**) (a) Laser-assisted bioprinting was explored for in-situ bioprinting of collagen-based bioinks for bone regeneration applications, (b) two different printed designs: a ring and a disk, and (c) disk printed geometry showed homogeneous regeneration throughout the defect, in contrast with the ring geometry, where regeneration is mainly observed at the periphery [126].

Following the extrusion printing process, additional crosslinking of collagen (mentioned in earlier sections) can tune the mechanical properties of the collagen scaffold as desired [41,46,58,59,67,68,70,117]. Additionally, the extrusion printing process was able to generate collagen-composite scaffolds loaded with bio-additives such as silk fibroin, β-TCP, HA via-freeze-drying process for bone tissue regeneration [46,59]. Extrusion bioprinting can be combined with inkjet bioprinting for a one-step process to produce cell-laden 3D skin tissue (Figure 2A) [118].

#### 3.2.2. Inkjet Printing

In inkjet bioprinting, biomaterials in a liquid state are loaded into a cartridge and deposited onto a substrate via droplets. The propulsion of droplets is achieved through pulses of pressure generated via thermal, acoustic or piezoelectric elements. The precise deposition of material is controlled by the dispensing system along the x, y-axis and print platform along the z-axis [18]. Through multiple print heads and cartridges, different material formulations can be combined. Additionally, as a nozzle-less systems, cell viability via inkjet bioprinting is higher compared to extrusion bioprinting. However, there is a material viscosity limit (10 mPa·s) for the inks printed due to the limited force generated to propel droplets onto the substrate [119]. Due to low-viscosity inks used in the system, additional processing steps are required to form 3D structures.

The viscosity limit of inkjet bioprinting restricts bioink formulations and bioink cell concentration. However, the self-assembly of collagen after printing allows it to be printed at low viscosity and crosslinked to produce cornea-like structures loaded with corneal stromal keratocytes (Figure 2B) [120]. Collagen ink blended with agarose in cell-laden printing gave rise to mesenchymal stem cells (MSCs) with a spread morphology, resulting in osteogenic differentiation [121]. Inkjet bioprinting was also used to generate collagen ink patterns onto which smooth muscle cells as well as neuronal cells were cultured, resulting in complex cellular patterns [122,123]. Additionally, inkjet bioprinting was applied to create in vitro cancer model microtissue arrays for drug testing and studying tumor progression [124]. Moreover, by controlling the thickness of the collagen gels printed via inkjet printing and seeding cells between the layers of the 3D construct, cell aggregates have been shown to fuse together, demonstrating potential for organ printing [125].

#### 3.2.3. Laser-Assisted Printing

In laser-assisted bioprinting (LAB), a layer of biomaterial is deposited onto a substrate via laser-induced forward transfer. A pulsed laser beam is focused on to a donor substrate coated with a laser-energy absorbing layer and a biomaterial layer. Energy absorbed by the donor would drive the biomaterial from the donor substrate onto the receiving substrate. The precise deposition of biomaterial is achieved by the movement of the donor substrate in the x, y axis and the receiving substrate in the x,y and z axis [18]. By coating the donor film with different materials and focusing the laser beam on different locations of the donor substrate for deposition, a heterogenous 3D structure can be obtained. This method of bioprinting, like extrusion bioprinting, also allows a large range of ink viscosities (1–300 mPa·s) [127]. It has the highest print resolution (10 µm) amongst additive bio-manufacturing methods and allows for a high concentration of cell loading [128]. However, preparation of a homogenous donor substrate for each cell type and biomaterials is time-consuming and may be difficult with multiple cells and material formulations.

Collagen-based inks are a suitable donor substrate due to cell biocompatibility and their potential for self-assembly and crosslinking. Laser-assisted bioprinting has been used to recreate skin substitutes [129,130] and corneal stroma-like tissue [131]. Additionally, in vivo bone regeneration was achieved by in situ printing of mesenchymal stromal cells using LAB (Figure 2C) [126]. 

#### 3.2.4. Stereolithography Printing

In stereolithography/digital light process (SLA/DLP) bioprinting, the ink is crosslinked by photopolymerisation. A reservoir of photo-sensitive ink is exposed to a predefined light pattern and crosslinked layer by layer onto a platform to produce a 3D structure [18]. The use of light patterns allow for high print resolution (50 µm) and accuracy [132]. Similar to nozzle free systems such as inkjet and laser-assisted bioprinting, SLA/DLP systems do not face clogging issues during printing. SLA accommodates inks with greater viscosities (<5 Pa) [133]. However, its restriction is the requirement for photopolymerisation crosslinking since not all materials are compatible for printing. Furthermore, photo-curing agents can be cytotoxic if residual components remain after printing [132]. Unlike previous methods, SLA/DLP bioprinting is unable to incorporate multiple ink formulations. 

While collagen can be crosslinked by UV irradiation, on its own, it cannot crosslink sufficiently fast for viable bioprinting. This necessitates functionalisation of collagen molecules. Typically, free amine groups in collagen are replaced with methacrylate groups which can participate in free radical polymerisation (methacrylation). Additionally, this functionalised collagen retains the ability to self-assemble into fibrillar structures upon neutralisation. Modified collagen has shown successful 3D photopatterning of hydrogels loaded with human mesenchymal stem cells [134].

To aid the reader, Table 1 has been provided to summarise applications of additive bioprinting methods for collagen biomaterials/biocomposites and bioinks (cell-laden). 

## 4. Regulatory Considerations and Challenges for Collagen Biomanufacturing

Currently, additive bioprinting methods have made significant progress using collagen biomaterials to repair severe skin wounds, regenerate cornea and (cranial) bone defects etc. In addition, precise spatial patterning of collagen biomaterials/biocomposites and bioinks (cell-laden biomaterials) can recapitulate complex tissue architecture for realistic in vitro testing. Being highly customisable, additive bioprinting will likely benefit the regeneration of hard- (bone) and soft- tissue trauma to kickstart tissue regeneration. Yet, regulatory and commercial aspects present a formidable bottleneck to their successful translation for therapy. 

Taking bone tissue engineering (BTE) as an example, even after 25 years of research and 100’s of $ millions of federal research (in the USA alone), clinical progress is limited. For example, 75% of spinal fusion procedures performed still use traditional grafting methods, suggesting that limited clinical benefits were derived from recent tissue engineering research [137]. Yet, certain approved therapeutics such as INFUSE^TM^ from Medtronic Plc reap >$750 Million in annual sales [138]. Thus, disparity between clinical translation success and failure is highly significant. This has been described as ‘the valley of death’ where promising technologies fail to transition into commercial usage. Past analysis suggests that translational failure can attributed to 2 stages: (i) between institutes of higher learning where fundamental research is carried out and industry, because promising ideas fail to attract sufficient funding to transition into industry and (ii) industry to clinical implementation—where funding is insufficient to complete human trials [139].

It might be instructive to consider regulations that govern the approval of therapeutics. In the USA, any prospective therapy would be assigned by the FDA to 3 centers: (i) regulate drugs (small-molecules, therapeutic proteins, antibodies and immune-modulators), (ii) regulate biological products (viruses, toxins, vaccines, blood components, cells, tissues gene vectors etc) (iii) medical devices. Separate offices of combination products, and cellular, tissue and gene therapies also have purview of the regulatory process. Further information is summarised in the review article by Pashuck & Stevens [138]. 

Broadly-speaking, therapies can be regulated as “drugs” or “devices” - a device does not “*achieve its primary intended purpose through chemical action (chemical reaction and/or intermolecular forces*)” [138]. These definitions have significant cost implications as new drug or biologic candidates cost approximately $850 million taking 5–10 years [138], whereas premarket approvals (PMAs) for new medical devices cost between $45–150 million and are typically completed within 5 years [138]. Notably, the PMA route is used for high-risk devices that require clinical safety and efficacy demonstrations involving approximately 1% of device applications. Accounting for a greater proportion, are lower-risk 510 (K) devices that utilise premarket notification (PMN) channels ($1–50 million to develop). These need to demonstrate equivalence or substantial equivalence to an existing marketed device [138]. Thus, acellular biomaterial scaffolds versus combination bioinks laden with cells and/or chemical agents (e.g., growth factors) are regulated very differently. 

One example is the role of collagen in the product Biobrane^®^ which reportedly acts relatively passively while supporting wound healing [140]. On the other hand, combination products may have biologics and drug ingredients which require oversight from the office of combination products and/or office of cellular, tissue and gene therapies [138]. For example, bioprinting skin constructs to repair severe wounds may require adding growth factors with chemical activity to assist wound regeneration. This potentially hinders swift and cost-effective regulatory approval [141]. The “rule of thumb” in product translation is that increasing product complexity correlates with the number and magnitude of challenges that need to be overcome before regulatory approval [140].

Furthermore, cGMP (current good manufacturing practice) is a requirement for mass production and ISO 10993 tests are required to assess biocompatibility. For cGMP, design history (allowable ranges of physical properties - material, geometry, porosity, mechanical etc) and device history (testing to demonstrate manufacturing design criteria was met) files are required, along with related auditing costs. Biocompatibility testing on large preclinical animals may cost a further $50 million prior to commencing human clinical trials [137]. One approach to cross this proverbial “valley of death” might involve developing technology in a modular manner. For example, development could begin with a minimally-modified biomaterial using the 510 (K) pathway to initiate revenue generation, before developing combination products suited for the PMA route. The likelihood of obtaining approval for the 2nd product with more complex features could be enhanced by the original (basic) product, because of its regulatory predicate [137]. 

A further consideration concerns differences between the EU and USA in regulating 3D bioprinted tissue engineering products. Whereas they may be considered biologics in USA, they are regulated as combined advanced therapy medicinal products (ATMPs) in EU. In general, the authors found that existing frameworks fail to address aspects of computer-aided 3D-bioprinting for additive manufacturing of customised tissue products [142]. They concluded, early and regular dialogue with regulatory authorities may alleviate these bottlenecks in manufacturing and quality development [142]. 

## 5. Concluding Remarks

As the most ubiquitous extracellular matrix material, collagen is an obvious candidate biomaterial with great promise for regenerative medicine. Collagen is a natural polymer with high biocompatibility, biodegradability and weak antigenicity [13]. Other benefits include: its evolutionary conservation [143]—suggesting it can be derived from many sources including (but not limited to) common commercial sources: rat tail, porcine tendon, bovine skin, fish skin etc. Thus, several xenogeneic acellular matrices have already obtained clinical approval [143]. Collagen is also extracted relatively easily, increasing the ease of availability. However, issues of ethical derivation and sustainability of collagen have arisen, which makes transgenic sources an attractive proposition [29]. Collagen is also a highly versatile biomaterial, denaturing into gelatin (and other derivatives), increasing crosslinking degree through chemical and physical means—rendering control over physical properties such as: mechanical stiffness, pore size and biodegradability. Its versatility extends to formulating biocomposites with inorganic and natural polymers to provide appropriate mechanical stiffness (e.g., PCL), gelation properties (e.g., alginates) etc. to develop suitable collagen bioinks and biomaterials for therapy.

Producing collagen-derived therapeutic and testing products with additive bioprinting methods provides significant benefits over non-additive production. Additive bioprinting exquisitely controls ink deposition, facilitating spatial patterning (mimicking the heterogeneity of skin dermis) [141], reproducibility, customisation, higher throughput, cost-effectiveness etc. [19]. On the other hand, non-additive methods like manual casting may limit product complexity and reproducibility, while electrospinning is limited in throughput and product complexity. These attractive attributes of additive bioprinting may significantly lower barriers to utilising collagen-based products in regenerative therapy and disease modelling etc. With increased process reproducibility, the inter-batch variability during manufacturing is likely to decrease, resulting in smaller tolerances reflected in its device master file (cGMP requirement). Therefore, strategic considerations of regulatory and cost issues in the application of additive bioprinting will help to ensure collagen biomaterials fulfil their tremendous potential in biomedicine and bioscience.

## Figures and Tables

**Table 1:** Applications of additive bioprinting methods for collagen-based inks.

Bioprinting Method	Collagen-Based Ink Formulation	Outcome	Ref.
Extrusion	Methacrylated type I collagen; Sodium alginate	Fabrication of structures that resembles native human corneal stroma with cell-laden bioink via extrusion bioprinting.	[116]
Extrusion	Collagen Type I; Alginic acid sodium salt from brown algae; CaCl_2_ solution	Core-sheath coaxial extrusion of alginate/collagen bioink with CaCl_2_ allows creation of scaffolds with low collagen centration despite its low viscosity.	[114]
Extrusion	Rat tail type I collagen; Gelatin (type A); Sodium alginate	Extrusion bioprinting of collagen scaffold via gelatin/alginate system with controllable degradation time based on amount of sodium citrate during incubation.	[113]
Extrusion	Type I collagen was extracted from tendons obtained from rat tails	Identified storage modulus as the best predictor of collagen bioink printability during deposition.	[117]
Extrusion	PureCol Purified Bovine Collagen Solution; Soldium alginate (low viscosity)	Fabrication of interwoven hard (PLLA) and soft (bioink) scaffolds which support cell attachment and proliferation using a modified desktop 3D printer.	[135]
Extrusion	Methacrylated COL I; Heprasil; Photoinitiator	Successful bioprinting of liver model. Printed primary hepatocytes retained function over 2 weeks exhibiting appropriate response to toxic drugs.	[41]
Extrusion	Lyophilized Atelo-collagen, Matrixen-PSP	Pre-set extrusion bioprinting technique is able to create heterogeneous, multicellular and multi-material structures which perform better than traditional bioprinting.	[112]
Extrusion	Collagen Type I extracted from rat tails; Pluronic^®^ F127	Fabrication of 3D constructs without chemical or photocrosslinking before and after printing via thermally-controlled extrusion.	[115]
Extrusion	Lyophilized sterile collagen, Viscoll	Formation of scaffolds which support spatial arrangement of tissue spheroids as well as support cell adhesion and proliferation.	[47]
Extrusion	Type-I collagen, Matrixen-PSP; Tannic acid	Fabrication of 3D porous structures which support cell migration and proliferation for long periods of culture. Determined optimal tannic acid crosslinking.	[67]
Extrusion	Collagen Type I; Sodium Alginate	Improved mechanical strength and bioactivity via the addition of collagen. Higher cartilage gene markers expressed, preservation of chondrocyte phenotype.	[42]
Extrusion	Type-1 collagen, Matrixen-PSP	Established a crosslinking process using tannic acid. High printed preosteoblast viability and well-defined pore size and strut dimensions for bone regeneration.	[68]
Extrusion	Type-I collagen, Matrixen-PSP; Decellularised extracellular matrix (dECM); Silk Fibroin(SF)	Hybrid collagen/dECM/SF scaffold with enhanced cellular activity and mechanical properties. Enhanced cell differentiation, mechanical properties, amenable for hard tissue regeneration.	[59]
Extrusion	Atelocollagen Type I powder	Novel self-assembly induced 3D printing to produce macro/nano porous collagen scaffolds with reasonable mechanical properties, excellent biocompatibility and mimicking native ECM.	[58]
Extrusion	Type-I collagen, Matrixen-PSP; Polycaprolactone (PCL); Hydroxyapatite (HA)/β-tricalcium-phosphate (TCP); Platelet-rich plasma(PRP)	Fabrication of collagen/PCL biocomposites loaded with bio-additives via 3D extrusion printing. Collagen/PCL biocomposites allow controlled release of HA/TCP bio-additives, which promote osteogenesis. PRP biocomposites demonstrate increased mineralisation.	[46]
Extrusion	Type-I collagen, Matrixen-PSP	Genipin crosslinking allowed fabrication of 3D cell-laden porous scaffold (Cellblock) with mechanical stability, pore size and osteogenic (bone tissue regeneration) potential.	[70]
Extrusion/Inkjet	Lyophilized collagen type 1 sponge derived from porcine skin	Development of a one-step process to produce a 3D human skin model with functional transwell system. Cost-effective compared to traditional transwell cultures.	[118]
Inkjet	Type I rat tail collagen; poly-d-lysine	Fabrication of neuron-adhesive patterns by printing cell-adhesive layers onto cell-repulsive substrates.	[123]
Inkjet	Collagen (Calf skin)	Cell aggregates printed between layers of collagen gels suitable for tissue engineering.	[125]
Inkjet	Collagen (rat-tail); collagen (calf skin)	Low-cost, high-throughput surface patterning with collagen and potentially, other proteins.	[122]
Inkjet	Collagen Type I	Fabrication of in vitro cancer microtissues via collagen inkjet printing. Four individual microtissues within one 96-well plate well, maintained for up to seven days.	[124]
Inkjet	Collagen: Type I rat tail collagen; Fibrinogen; Thrombin	Collagen bioinks and Fibrin/Collagen bioinks unsuitable for in situ inkjet bioprinting.	[136]
Inkjet	Type I acidic collagen; Agarose (low gelling temperature)	Fabrication of 3D corneal stromal structure with optically properties similar to native corneal stroma. Potential as a clinical or experimental model.	[120]
Inkjet	Acidic collagen solution; Agarose (low gelling temperature)	MSC branching, spreading and osteogenic differentiation controlled by collagen concentration; Osteogenic potential (bone tissue engineering).	[121]
Laser-assisted	Collagen Type I (Rat-tail)	Fabrication of cell-laden skin tissue using laser-assisted bioprinting, in vivo potential. Skin tissues consist of: a base matriderm layer, 20 layers of fibroblast and 20 layers of keratinocytes.	[130]
Laser-assisted	Collagen (Rat-tail)	Multicellular collagen skin tissue constructs printed using laser-assisted bioprinting. Keratinocyte and fibroblast layers did not intermix after 10 days. Mimics tissue-specific functions (e.g., gap-junction).	[129]
Laser-assisted	Type I collagen (rat) solution; Nano hydroxyapatite (nHA)	In situ printing of cell-laden collagen-based ink via laser assisted bioprinting allow bone regeneration (mouse calvaria defect model). Contact free printing method is sterile with clinical potential.	[126]
Laser-assisted	OptiCol™ human Col I; Ethylenediaminetetraacetic acid (EDTA) human female AB blood plasma; Thrombin from human plasma	Fabrication of 3D cornea tissue using novel human protein bioinks via laser assisted bioprinting. Novel bioink is biocompatible, without requiring additional crosslinking. First study to demonstrate laser-assisted bioprinting for corneal applications using human stem cells.	[131]
Stereolithography (SLA)	Collagen methacrylamide(CMA) synthesized using Type-I collagen; Irgacure (I2959)	Free-form photolithographic fabrication; photopatterned hydrogels retain structure after 24 h. CMA retains native collagen self-assembling properties; hydrogels biocompatible in vivo.	[134]
